# Status of Two Species of Lac Insects in the Genus Kerria from China Based on Morphological, Cellular, and Molecular Evidence

**DOI:** 10.1673/031.011.10601

**Published:** 2011-08-22

**Authors:** Xiaoming Chen, Hang Chen, Ying Feng, Rui He, Zixiang Yang

**Affiliations:** ^1^ Research Institute of Resource Insect, Chinese Academy of Forestry, Kunming 650224, China; ^2^ Key Laboratory of Cultivating and Utilization of Resources Insects of State Forestry Administration, Kunming 650224, China

**Keywords:** cladistic analysis, EF1αgene, karyotype, *Kerria ruralis*, *Kerria yunnanensis*

## Abstract

The taxonomic status of the Chinese lac insects *Kerria yunnanensis* (Ou and Hong) (Hemiptera: Kerridae) and *K. ruralis* (Wang, Yao, Teiu and Liang) were analyzed in this paper by comparing morphological, cellular, and molecular data. Cladistic analysis showed *K. yunnanensis* and *K. ruralis* to be distinct from other *Kerria* species such as *K. lacca* and *K. chinensis*. The karyotype of *K. yunnanensis* was 3A and the chromosome structure was K = 6m + 2sm + 10T, while in *K. ruralis* the karyotype was 3B and the chromosome structure was K = 8m + 10T. *Kerria ruralis* and *K. yunnanensis* had the closest relationship among species in the genus as they had the most similar karyotype homology. Based on the karyotype analysis, *K. sindica* and *K. lacca* formed a sister group with *K. ruralis* and *K. yunnanensis*. *Kerria pusana* and *K. nepalensis* were clustered as a sister branch, indicating the close relationship of these taxa. The karyotype of *K. chinensis* was however, different from the other six species and formed a separate branch. RAPD analysis also showed that *K. yunnanensis* and *K. ruralis* had distinct differences from other species of *Kerria*, although they did not form sister taxa. Molecular analysis based on the EF1α gene using ML, MP, and Mr. Bayes' methods indicated that seven species of lac insects cluster in two major groups. In group 1, *K. sindica* and *K. lacca* formed a sister clade and were primitive members of the genus. In group 2, *K. chinensis* formed the earliest diverging branch followed by *K. ruralis*. *Kerria yunnanensis* was the next to diverge followed by the cluster containing *K. pusana* and *K. nepalensis*. Hybridization testing showed that crosses neither between *K. yunnanensis* and *K. sindica*, nor between *K. yunnanensis* and *K. lacca* could produce first generation larvae. This was indicative that *K. yunnanensis* had a distant genetic relationship from the other species. Morphological, cellular, molecular, and hybridization results confirmed the independent status of the Chinese endemic species *K. yunnanensis* and *K. ruralis*. *Kerria ruralis* was genetically closely related to *K. yunnanensis*, but relatively far from *K. lacca*. The main commercial species in China was *K. yunnanensis*, while in Thailand it was *K. chinensis*. The commercial species in Myanmar included *K. nepalensis* and *K. pusana*, the latter being most widely used in lac production.

## Introduction

Lac insects are scale insects belonging to the family Kerriidae. These insects are considered to be of great economic value. Shellac that is produced by the lac insects of the genus *Kerria* is widely used in chemicals, electronics, military, food, and other industries (Chen 2005). The family Kerriidae contains nine genera and approximately 100 species (Ben-Dov 2002), while the genus *Kerria*, contains 19 species which have mainly been used in the lac industry. *Kerria chinensis* (Mahdihassan) (Hemiptera: Kerridae), *K. ruralis* (Wang, Yao, Teiu and Liang), *K. yunnanensis* (Ou and Hong), *K. fici* (Green), and *K. greeni* (Chamberlin) are reported from China, with *K. yunnanensis* and *K. ruralis* being endemic (Chamberlin 1923; Mahdihassan 1923; Varshney 1976, 1984; Wang 1982; Ou and Hong 1990; Chen 2005).

Shellac is mainly produced in China, India, Myanmar, Laos, and Thailand. The main commercial species of lac insect in India is *K. lacca*, while in Thailand it is *K. chinensis* (Chen 2005). There has long been controversy in the identification of commercial lac species in China. Lac insects in China were firstly named *Laccifer lacca* in the 1950s (Liu 1957, 1959). In 1982, the genus *Laccifer* was synonymized with *Kerria* and this has generally been accepted in accordance with taxonomic studies in India (Varshney 1976). The fresh collections in China were compared with *K. lacca* and several differences were found which resulted in the naming of the species *K. yunnanensis* (Ou and Hong 1990). Also, the commercial species in Thailand was regarded as *K. yunnanensis* (Ou and Hong 1990). However, lack of comparison with *K. chinensis* meant there were still certain aspects in need of clarification; and the Chinese commercial species was tentatively named as *K. chinensis* (Chen et al. 1992, 1998). Another endemic species in China was found and named *K. ruralis* (Wang et al. 1982), and this species showed a close relationship with *K. fici* and *K. lacca*. *Kerria fici* was first recorded in China (Chamberlin 1923), but has not been found there since 1930s (Chen et al. 2008). *Kerria greeni* was reported to occur in Yunnan and Fujian Provinces of China (Chen 2005).

The purpose of this study is to clarify the taxonomic status of two species of *Kerria* in China. Fresh insects were collected from their host in the locations according the first record in publication. The two species were compared morphologically at the cellular level by studying their karyotypes, RAPD reactions were performed, they were sequenced with EF1α genes and hybridization to establish their identifications, and their relationship with other species in genus *Kerria* was analyzed.

## Materials and Methods

### Materials

The following samples used in this study are listed in Table 1. *Kerria yunnanensis* was identified by Ou Bingrong and Hong Guangji. *K. ruralis* was identified by Wang Ziqing. Both of them were collected in Yunnan, China. *K. chinensis* was collected in Thailand. *K. pusana* and *K. nepalensis* were collected in Burma. The three species were identified by authors after a discussion with Dr. Xie Yinping, who is an expert of scale insect in Shanxi University, China. *K. sindica* was identified by Mahdihassan S and collected from Pakistan. *K. lacca* was identified and presented by Indian Institute of Natural Resins and Gums.

### Cladistic analysis based on morphology

Permanent slides of mature adult female lac insects were prepared by the method used by Chen et al. (2008). Twenty-two apomorphies which included brachia, anal tubercles, dorsal spine, anterior spiracles, posterior spiracles, and perivulvar pore clusters were selected as morphological characters. The average values for thirty individuals of each species were measured. Phylogenetic analyses were performed under maximum parsimony (Nei and Kumar 2000) and analyses conducted using PAUP* 4.0b10 (Swofford 2001). To test for nodal reliabilities, heuristic bootstrap analyses (Felsenstein 1985; 1000 replicates) were applied with groups appearing in 50% or more of the trees in bootstrap analysis.

### Karyotype analysis

Microphotographs of chromosomes in different species were taken with a Nikon E800 optical system using the air-dried method (Chen et al. 2007) and karyotypic parameters were measured by IM50 software (Leica Ltd. 1992). Karyotype analysis was performed according to the standard method (Leven 1964; Stebbins 1971; Guo 1972). Phylogenetic relationship of lac insects were studied to built the dendrogram clustered using UPGMA by applying karyotype resemblance-near coefficients (λ) and the evolution distance with specific software (Li et al. 2005).

### RAPD analysis

The technique of random amplified polymorphic DNA (RAPD) was used to study the relationships of 12 populations from 7 species of *Kerria*. The genetic distance and identity among species were generated by POPGENE32 (Yeh and Boyle 1997). The molecular dendrogram was constructed based on Nei's genetic distance by MEG A3 using the UPGMA method (Kumar et al. 2004).

### PCR and Sequencing

Total genomic DNA was isolated from whole insect body using a standard proteinase K, phenol/chloroform extraction technique (Marchant 1988; Tian 1999). EF1α genes were amplified by polymerase chain reaction (PCR) using primer pair Forward (5′-ATGTGAGCAGTGTGGCAATCCAA-3≈) and Reverse (5′-GAACGTGAACGTGGTATCAC -3′) (Palumbi 1996).

DNA amplifications were carried out in the Bio-RAD MyCycler thermal cycler. Amplification cycles were as follows: 95° C for 4 min as initial denaturation step; 35 cycles of 64° C denaturation for 1 min, 72° C annealing for 2 min, 72° C extension for 7 min, and ended by cooling at 4° C. The resulting sequences were assembled using Bioedit version 7.0.5.3 (Hall 1999) then deposited in GenBank (Accession numbers EU781492 - EU781498), and are listed in Table 1.

Alignments for the individual gene data matrices were generated using similarity calculated at the nucleotide level with ClustalW version 1.81 of Lasergene DNAstar software package (Thompson et al. 1997). Bayesian phylogenetic inference was used to estimate the tree topology by Mr. Bayes 3.1.2 (Huelsenbeck and Ronquist 2001). ML trees were generated based on hierarchical likelihood ratio test (hLRT test) estimated by Modeltest 3.06. Statistical support for each node was evaluated by bootstrap analysis (Felsenstein 1985). Parsimony analyses were conducted in PAUP 4.0b software (beta 10^th^ version, Swofford 2001). Tree visualization and drawing were carried out with Tree View version 1.5.2 (Page 1996).

### Hybridization test

All male insects were manually removed from twigs harboring second instar larvae and the remaining females were covered by a synthetic net sleeve (80 mesh), which protected the insects from attack from parasitoids and predators. When the females in the sleeves developed into adults, they were copulated with males chosen from other species. The female insects not copulating were treated as controls.

## Results

### Morphological Diagnosis

***Kerria yunnanensis* (Ou and Hong) 1990: 15.**
Adult female: 1.04–1.9 mm long, 0.69–1.38 mm wide, globe-like body, dark reddish brown. Anal tubercle heavily sclerotized with 0.08–0.38 mm long and 0.06–0.36 mm wide, nearly quadrate, apparently two-segmented and harboring 6–13 anal ring setae about 0.18–0.27 mm long. Branchial tube less than 0.09 mm high, brachial plate with crater about 0.10–0.15 mm long, 0.08–0.14 mm wide, and 0.03–0.05 mm^2^ in the center. Dimples in crater are formed by the brachial pores, numbers vary from 8 to 15. Anterior spiracles are situated 0.02–0.11 mm to brachial plates with 0.18–0.3 lmm in length and 0.10–0.15mm in width, inside the keratinization trail is inconspicuous and less than 0.22 mm. Dorsal spines are found between the brachia and anal tubercle, and have two parts: a stout pedicel about 0.01–0.11 mm long and 0.04–0.05 mm wide and a conspicuous scletotized spine averaged 0.10–0.23 mm in length. Perivulvar pore clusters originate circularly near the anal tubercle. Mouthparts have a labium about 0.31–1.40 mm long, 0.11–0.21 mm wide with inconspicuous segmentation and a pair of post oral lobes 0.03–0.12 mm wide just behind the mouth.
Holotype: ♀, paratypes, 8 ♀♀, 4 May 1987, Yunnan, P.R. China (Ou and Hong 1990).
Biological characteristics: Bi-voltine, summer (May–October) and winter crops (October-the next May). Life history of females and males are list in Table 1 and Table 2, respectively.
Host: *Dalbergia obtusifolia* (Baker) Prain.
Distribution: Subtropical areas of Pu'er and Lincang of Yunnan Province, P.R. China.

***Kerria ruralis* Wang 1982: 53.**
Adult female: Length on slide 1.02–2.3 mm and 0.58–1.27 mm wide, globe-like body, two body color types, i.e. dark reddish brown or yellow. Anal tubercle heavily sclerotized, 0.06–0.36 mm long and 0.21–0.37mm wide, apparently two-segmented and consist 1–13 anal ring setae about 0.17–0.31 mm long. Brachial tube less than 0.09 mm long, brachial plate with a crater about 0.08–0.13 mm long, 0.06–0.11 mm wide and 0.03–0.04 mm^2^ in the center. Numbers of pores in crater vary from 4 to 11. Anterior spiracles are situated 0.02–0.11 mm to brachia 0.18–0.26 mm long and 0.10–0.15 mm wide, inside the inconspicuous keratinization trail is less than 0.12 mm. Dorsal spine heavily sclerotized 0.10–0.23 mm long; pedicel of dorsal spine 0.02–0.16 mm long, 0.04–0.13 mm wide. Perivulvar pore clusters circular, present near anal tubercle. Mouthparts with a labium about 0.21–0.82 mm long, 0.12–0.19 mm wide, with inconspicuous segmentation and a pair of post oral lobes, each 0.06–0.14 mm wide, behind the mouthparts.
Holotype: ♀, paratypes, 7 ♀♀, 10 June 1969, Yunnan, P.R. China (Wang et al. 1982)
Biological characteristics: Bi-voltine, summer (March–July) and winter crops (August-the next March). Life history of female and male were list in Table 3 and Table 4, respectively. Two body color types, i.e. red and yellow. The ratio of red:yellow is about 12:1.
Host: *Mallotus philippinensis* (Lam.) Muell.
Distribution: Tropical and subtropical areas of Pu'er and Xishuangbanna of Yunnan Province, China.

### Cladistic Analysis

**Figure 1.**
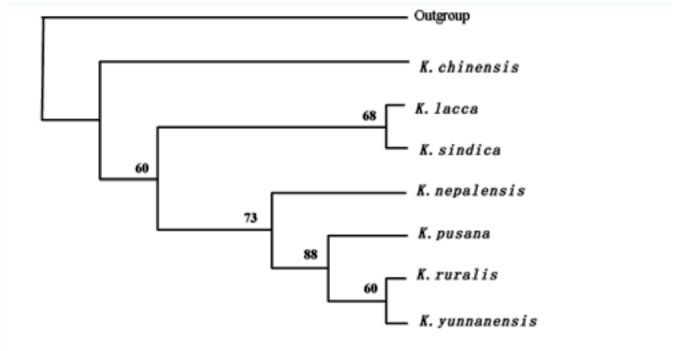
Phylogenetic hypotheses among seven species of lac insects based on the morphological characters by the MP method (The numbers above branches are bootstrap values, Chen et *al*., 2008). The aphid *Stomaphis japonica* (Hemiptera: Aphididae) is the outgroup. High quality figures are available online.

Based on the morphological study of twentytwo apomorphies (Table 6), *K. ruralis* and *K. yunnanensis* should belong to different categories with obvious differences from other species such as *K. lacca* and *K. yunnanensis* (Chen et al. 2008). The phylogenetic relationships among seven species of lac insects using a cladistic approach showed that *K. chinensis* was the earliest diverging member in case of its special morphological characters. *Kerria ruralis* and *K. yunnanensis* were the newest species with similarity in morphology and form a sister group. Figure 1 shows the results. These specimens and Permanent slides were deposited in Research Institute of Resource Insect of China.

### Karyotype analysis

**Figure 2.**
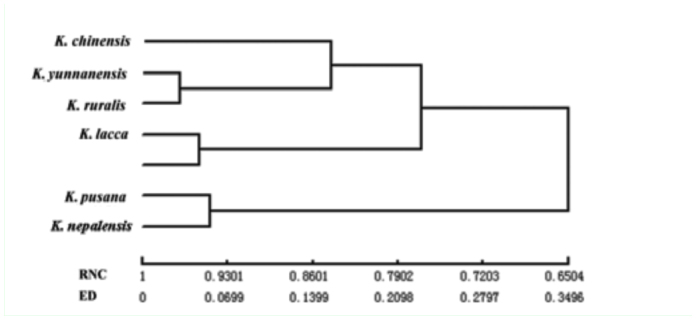
Dendrogram of seven species of lac insect based on the karyotype resemblance -near coefficients and evolutionary distance. RNC = Resemblance -near coefficients. ED = Evolutionary distance. High quality figures are available online.

The chromosomes of seven species including *K. yunnanensis, K. ruralis*, *K. lacca*, *K. chinensis*, *K. sindica, K. pusana*, and *K. nepalensis* were composed of metacentric (or sub-metacentric) and telocentric chromosomes, showing the consistency of the genus while there were four kinds of chromosomes structure shape: K = 10m + 8T, K = 8m (6m + 2sm) + 10T, K = 6m (4m + 2sm) + 12T, K = 4m + 14T and the data are shown in Table 7 (Chen et al. 2007). The chromosomes of *K. yunnanensis* are made of six metacentric (or sub-metacentric) and ten telocentric chromosomes. And *K. ruralis* were formed with eight metacentric and ten telocentric chromosomes. Both differ from other species and have a certain degree of uniqueness. Differences of interspecific relationship were reflected in the centromere position of chromosomes. The cluster analysis method of karyotype resemblance-near coefficient indicated *K. ruralis* and *K. yunnanensis* had the highest identity in karyotype (0.9688) and nearest distance in evolution (0.0317), which showed they were the latest species of the seven grouped in the dendrogram (Table 8, Figure 2).

### RAPD analysis

**Figure 3.**
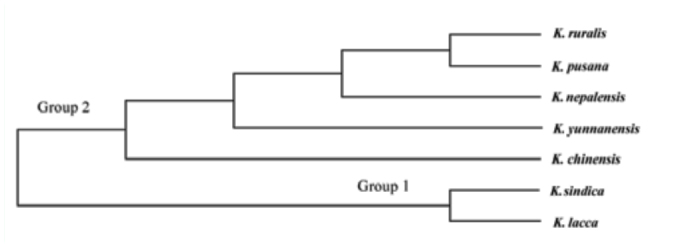
Dendrogram of lac insect among seven species based on genetic distances using the method of UPGMA. High quality figures are available online.

The results of RAPD analysis showed the genetic distance inter-species were 0.1854–0.7917, in which the average genetic distance among species was 0.4430 (Table 9) (Chen et al. 2006). The value of genetic distance between *K. yunnanensis* and *K. chinensis*, and *K. lacca* were 0.6297 and 0.5789, respectively; obviously for different categories. The results of UPGMA showed that the seven species could be divided into two natural groups (Figure 3). In group 1, *K. sindica* and *K. lacca* had the closest relationship and were sister taxa. In group 2, *K. nepalensis* was the nearest taxon to the *K. ruralis* and *K. pusana* sister group followed by *K. yunnanensis*. *K. chinensis* was the earliest diverging member of this group and is placed as the base branch in the group.

### Phylogeny of EF1α gene

**Figure 4.**
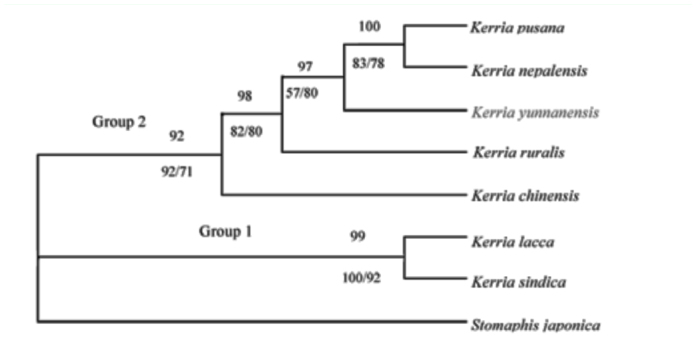
Majority-rule consensus tree resulting from Bayesian analysis of EF1α gene (model =GTR+G) from seven species of lac insects and the aphid *Stomaphis japonica* (Hemiptera: Aphididae) as outgroup. Branches represented are based on the maximum likelihood topology. Numbers above internodes indicate Bayesian posterior probabilities; numbers below internodes indicate nonparametric bootstrap proportions for the parsimony analysis (left) and likelihood analysis (right). Thickened branches indicate Bayesian posterior probabilities ≥95%. High quality figures are available online.

The phylogenetic trees are based on EF1α gene data of seven lac species, the topologies made by using of MP, ML, and Bayesian methods were similar with different bootstrap values varying from moderate to high for different branches (Figure 4). In the MP tree, all the branches received high bootstrap values (>70%) except the weakly supported branch of *K. yunnanensis* (57%), in which MP tree length = 377, CI = 0.976, RI = 0.813, and RC = 0.793 Using the maximum likelihood (ML) to build the phylogenetic tree, the GTR + G model was selected as the best model for phylogenetic analysis in accordance with the hLRT test (-InL = 2913.3172). The support value of all branches was in excess of 70%. Bayesian analysis results were consistent with the systematic relationships of MP and ML trees. Except the branch of *K. chinensis*, which was 92%, other branches all received high posterior probability (PP > 95%).

The seven lac species clustered into two distinct clades (group 1 and 2 in Figure 4), one containing *K. sindica* and *K. lacca* (Bayes, 99%; MP, 100%; ML, 100%) and the other containing the remain five species (Bayes, 92%; MP, 92%; ML, 71%). In group 1, *K. sindica* and *K. lacca* had a close relationship and were sister taxon. In group 2, *K. nepalensis* and *K. pusana* formed a sister branch, showing a close relationship, and indicated that they were the most recently evolved species of the seven. *K. chinensis* was the earliest diverging member of group 2, and has a distant relationship with the others. *K. yunnanensis* and *K. ruralis* were earlier evolved species, but they diverge later than *K. chinensis. K. pusana* and *K. nepalensis* were the most recently evolved species of the taxon, forming a close sister group with high support value (Bayes, 100%; MP, 83%; ML, 78%).

### Hybridization test

Hybridization tests showed that the mating between *K. lacca* and *K. sindica* produced first filial generation, indicating the close genetic relationship of the two species. However, crossbreeding between *K. yunnanensis* and *K. sindica*, or that of *K. yunnanensis* and *K. lacca*, did not produce any first filial generation, which indicated *K. yunnanensis* had distant relationship to *K. sindica* and *K. lacca* (Table 10) (Chen et al. 1992). Five kinds of hybridization could not produce normal offspring (Table 10), which indicated that three populations belong to separate species with the existence of reproductive isolation among them.

## Discussion

In this study, *K. ruralis* and *K. yunnanensis* should belong to different categories, with obvious differences from other five species on the morphological characters. The results were in accord with the study of morphological characteristics by using scanning electron microscopy, which confirmed lac commercial species in China was a new species with clear differences from *K. lacca* and nominated as *K. yunnanensis* (Ou and Hong 1990, 1991). And the two species also differ from other species with the unique chromosomes structure as K = 8m (6m + 2sm) + 10T (Table 7). In RAPD analysis, the genetic distance between *K. ruralis* and *K. chinensis*, and *K. lacca* were 0.5604 and 0.5110, respectively (Table 9), both higher than the average value of inter-species, which proved the three individuals should be classified into different species. This was in contrast to the idea that lac production species in China were the same as *K. lacca* in India (Liu 1957, 1959). On the phylogenetic trees based on EF1α gene data, the branches of *K. yunnanensis* and *K. ruralis* were highly supported with MP, ML, and Bayes analysis in diverging with other species (Figure 4). Results of hybridization also confirmed that the species used for lac production in China was not *K. lacca*, but *K. yunnanensis* (Chen et al 1992), which is the commercial species in China that is very different from *K. chinensis*, the commercial species of Thailand.

Morphology, cytology, and molecular biology evidence consistently indicated *K. yunnanensis* and *K. ruralis*, the two Chinese endemic species, had significant differences with the other five species examined in this study. The relationships of the seven species were basically consistent, with a few phylogenetic positions being incomplete. However, with no matter which methods, *K. lacca* and *K. sindica,* and *K. pusana* and *K. nepalensis*, always clustered together and formed two sister groups indicating a close genetic relationship between them. With the exception of RAPD, *K. nepalensis* and *K. pusana* always clustered together, indicating a close genetic relationship. *K. chinensis* was rather special and always stood alone in a separate branch.

The phylogenetic analysis also found that species distributed in similar ecological environments usually clustered indicating a near relationship. *K. lacca* and *K. sindica* were both distributed in tropical regions, in which *K. lacca* was originally found (India, Pakistan, Nepal, Bangladesh, and Sri Lanka). *K. sindica* was originally located in Sindh Province of Pakistan, mainly located in the lower reaches of the India River in Hyderabad and Karachi, with an annual temperature above 24° C and an annual rainfall of 250–400 mm, which both indicate a tropical monsoon climate (Table 11). The phylogenetic tree (Figure 4) showed *K. sindica* and *K. lacca* had the closest relationship and were sister taxa with high support values (Bayes, 99%; MP, 100%; ML, 92%), in the most basal branch of seven species.

*Kerria chinensis* was found in the north and northeastern of Thailand, where the average annual temperature was 22–28° C and the annual precipitation is 1300–1900 mm (Table 11). The environment was different from that of other species, and this taxon stood alone as a separate branch (Figure 4). *K. yunnanensis* and *K. ruralis* diverged later than *K. chinensis* in this group. While *K. pusana* and *K. nepalensis* were the most recent of the genus, forming a close sister group with high support values (Bayes, 100%; MP, 83%; ML, 78%). These four more advanced species were mainly distributed in edge of south subtropical and the north tropical regions.

Among the four species of group 2 in Figure 4, *K. yunnanensis* was found in semi-arid and semi-humid area of Southern subtropical zone, with 600–1500 m elevation and 18–20° C average annual temperature. *K. ruralis* occured in Xishuangbanna, Yunnan Province in the type humid subtropical climate, close to tropical north border, where the average annual temperature was 19–21° C and the annual precipitation was 1200–1700 mm. *K. pusana* and *K. nepalensis* were distributed in different altitudes in Myanmar. *K. pusana* distributed in 800–1400 m altitude area of Taunggyi, Lashio, Meimiao, which belonged to subtropical climate with average annual temperature of 19–20° C and 1200–1500 mm average annual rainfall. While *K. nepalensis* was located at low elevations in south Mandalay, about 200 m above sea level, in a tropical monsoon climate with annual average temperature of 23–29° C and an average of 800–1000 mm annual precipitation.

Based on the above results we concluded that the climate was possibly the main factor that deduced the inter-species diversity of genus *Kerria* in different environment. The unique taxonomic statuses of two species in China were proved by comparison in morphological, cellular, and molecular levels with other species in genus *Kerria.* Evidence from this study showed the commercial species in China was *K. yunnanensis* and this species was very different from *K. chinensis*, the commercial species of Thailand. *K. ruralis* and *K. lacca*, the main commercial species in India, differed in morphological characters, ecological location, chromosome structure, and genetic material. The two species of lac production in Myanmar were *K. pusana* and *K. nepalensis*, of which, *K. pusana* is most widely used in lac industry. The main commercial species of lac insect in various countries, with differences in climate and environment, belong to different species.

**Table 1.**
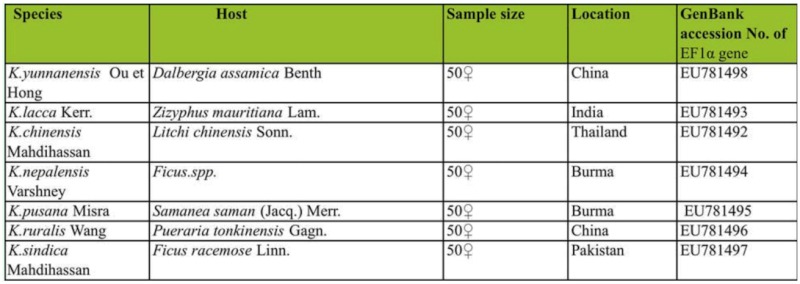
Basic information of the samples sevev species of lac insect.

**Table 2.**
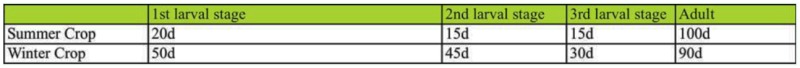
Life history of *Kerria yunanensis* (Female)

**Table 3.**
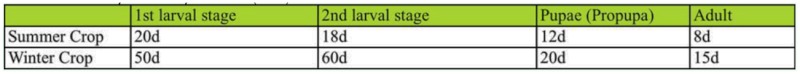
Life history of *Kerria yunanensis* (Male)

**Table 4.**
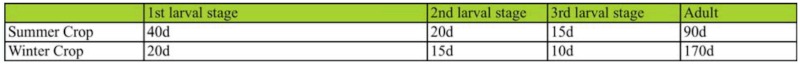
Life history of *Kerria ruralis* (Female)

**Table 5.**
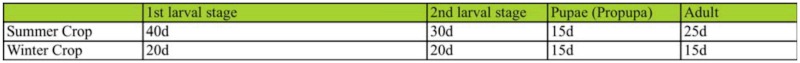
Life history of *Kerria ruralis* (Male)

**Table 6.**
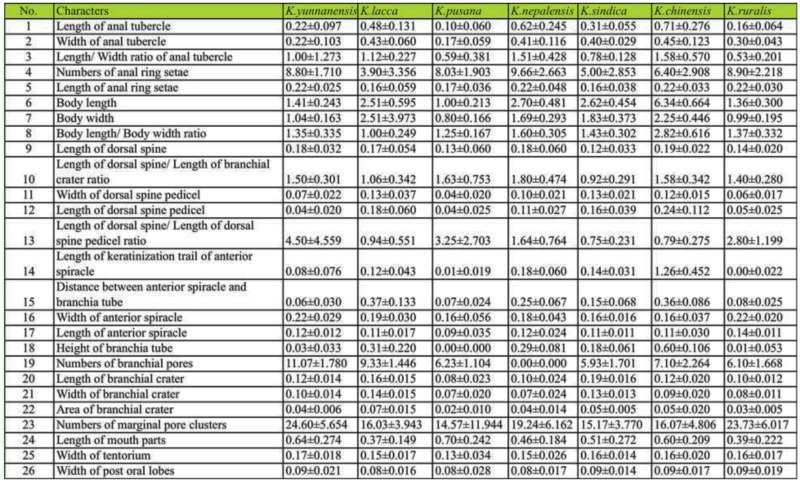
The numerical value of twenty-two morphological characters of seven species (mm)

**Table 7.**
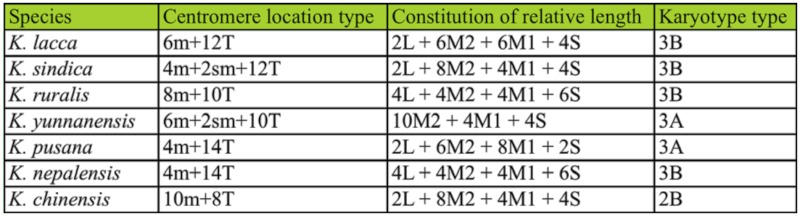
The comparison of karyotypic analysis among seven species of lac insect

**Table 8.**
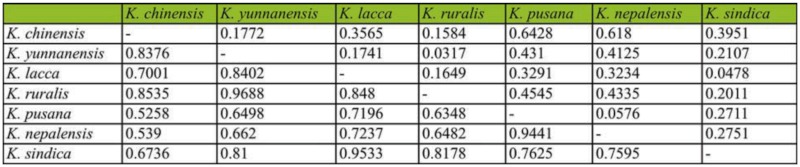
Karyotype resemblance-near coefficients (below diagonal) and evolutionary distance (above diagonal) in seven species of lac insects

**Table 9.**
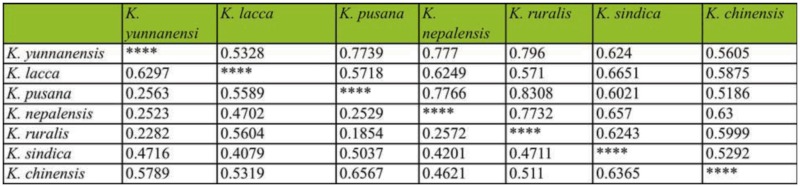
Nei's genetic distance (below diagonal) and genetic identity (above diagonal) among seven species of lac insect generated by RAPD

**Table 10.**
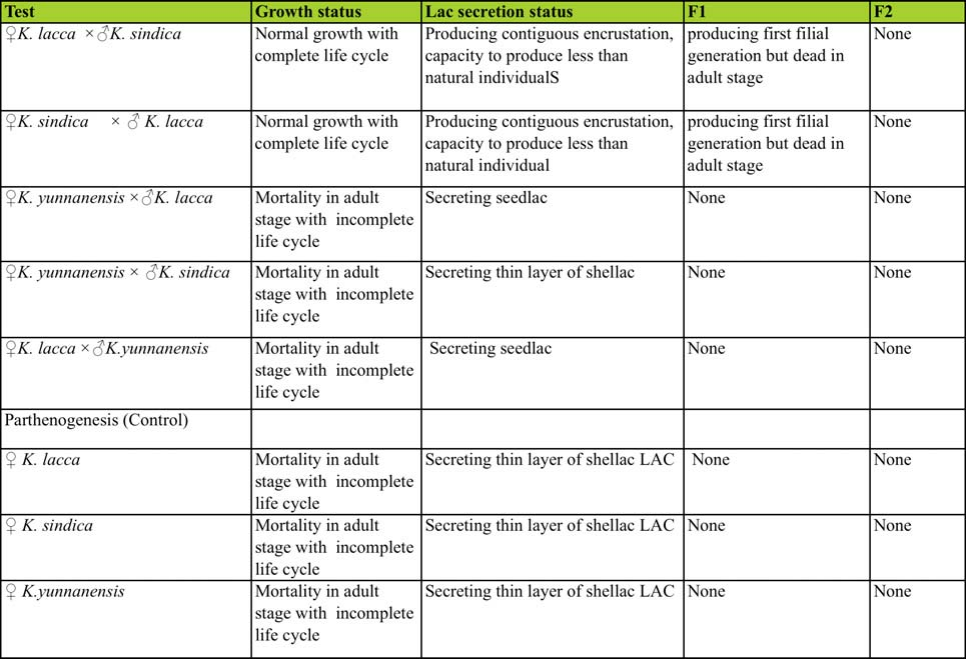
Hybridization test of three species of Lac insects

**Table 11.**
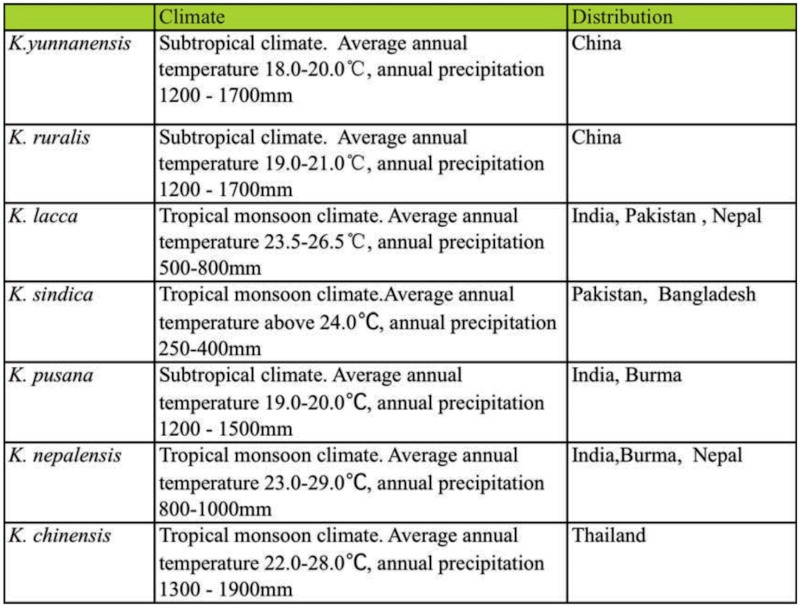
Environment comparison of seven species of Lac insects
